# Annotation of 1350 Common Genetic Variants of the 19 ALDH Multigene Family from Global Human Genome Aggregation Database (gnomAD)

**DOI:** 10.3390/biom11101423

**Published:** 2021-09-29

**Authors:** Che-Hong Chen, Benjamin R. Kraemer, Lucia Lee, Daria Mochly-Rosen

**Affiliations:** Department of Chemical and Systems Biology, Stanford University School of Medicine, Stanford, CA 94305, USA; bkraemer@stanford.edu (B.R.K.); lucialee@stanford.edu (L.L.)

**Keywords:** aldehyde dehydrogenase, ALDH, aldehyde, genetic variants, gnomAD

## Abstract

Human aldehyde dehydrogenase (ALDH) is a multigene family with 19 functional members encoding a class of diverse but important enzymes for detoxification or biotransformation of different endogenous and exogenous aldehyde substrates. Genetic mutations in the ALDH genes can cause the accumulation of toxic aldehydes and abnormal carbonyl metabolism and serious human pathologies. However, the physiological functions and substrate specificity of many ALDH genes are still unknown. Although many genetic variants of the ALDH gene family exist in human populations, their phenotype or clinical consequences have not been determined. Using the most comprehensive global human Genome Aggregation Database, gnomAD, we annotated here 1350 common variants in the 19 ALDH genes. These 1350 common variants represent all known genetic polymorphisms with a variant allele frequency of ≥0.1% (or an expected occurrence of ≥1 carrier per 500 individuals) in any of the seven major ethnic groups recorded by gnomAD. We detailed 13 types of DNA sequence variants, their genomic positions, SNP ID numbers, and allele frequencies among the seven major ethnic groups worldwide for each of the 19 ALDH genes. For the 313 missense variants identified in the gnomAD, we used two software algorithms, Polymorphism Phenotyping (PolyPhen) and Sorting Intolerant From Tolerant (SIFT), to predict the consequences of the variants on the structure and function of the enzyme. Finally, gene constraint analysis was used to predict how well genetic mutations were tolerated by selection forces for each of the ALDH genes in humans. Based on the ratio of observed and expected variant numbers in gnomAD, the three ALDH1A gene members, ALDH1A1, ALDH1A2, and ALDH1A3, appeared to have the lowest tolerance for loss-of-function mutations as compared to the other ALDH genes (# observed/# expected ratio 0.15–0.26). These analyses suggest that the ALDH1A1, ALDH1A2, and ALDH1A3 enzymes may serve a more essential function as compared with the other ALDH enzymes; functional loss mutations are much less common in healthy human populations than expected. This informatic analysis may assist the research community in determining the physiological function of ALDH isozymes and associate common variants with clinical phenotypes.

## 1. Introduction

The human genome contains an estimated 20,000–25,000 protein-coding genes [1], and 19 genes encode functional ALDH isozymes. Aldehyde dehydrogenases (ALDH, EC 1.2.1.3) are a group of non-P450 aldehyde oxidizing enzymes. The gene family plays a key role in metabolizing a diverse group of biogenic and xenogenic reactive aldehydes in the human body. Aldehydes are strong electrophiles, highly reactive, and are participants of many important physiological reactions for detoxification, metabolism, and biotransformation of amino acids, lipids, hormones, neurotransmitters, carbohydrates, and drugs [2]. Aldehydes are not only relatively long-lived reactive molecules compared to reactive oxygen species (ROS), but can diffuse and react with different cellular components and macromolecules to form Schiff’s bases or Michael adducts to incur damages to cellular components by crosslinking proteins and DNA [3]. Therefore, elevated aldehyde levels and accumulation of aldehydic adducts have been implicated in many human diseases [4,5]. Examples of common reactive or toxic aldehydes from different sources are acetaldehyde from alcohol drinking [6]; acetaldehyde and acrolein from air pollution and cigarette smoke [7]; acrolein and chloroacetadehyde as metabolites of oxazaphosphorine cancer drugs [8]; 3,4-dihydroxyphenylacetaldehyde (DOPAL), 3,4-dihydroxyphenylglycolaldehyde (DOPEGL) as intermediate metabolites of neurotransmitters, dopamine, and norepinephrine, respectively [5]; retinal as a metabolite from oxidation of retinol to produce retinoic acid, an important signaling molecule essential for embryogenesis and development [9,10]; malondialdehyde (MDA) and 4-hydroxynoneal (4HNE) from cellular lipid membrane peroxidation under oxidative stress [11]; α-aminoadipic semialdehyde in lysine metabolism [12], and many others. Biogenic and xenogenic aldehydes relevant to human exposure exist in various chemical structures, including short-chain, long-chain aliphatic aldehydes, aromatic aldehydes, di-aldehydes, and α,β-unsaturated aldehydes. There are more than 200 different aldehyde species just from the oxidative degradation of cellular membrane lipids alone [11]. The 19 human ALDH isozymes have evolved to recognize and metabolize different aldehydes with a high degree of specificity for certain groups of substrates for each isozyme, but also with overlapping specificities for substrate groups within the family [3,13]. Several extensive reviews on the evolution, chromosomal location, subcellular location, function, enzyme kinetics, substrate specificity have been published [2,3]. Although the primary role of the ALDH protein family is to oxidize different aldehyde substrates, ALDHs are also known to display non-catalytic functions for the generation and homeostasis of NAD(P)/NAD(P)H [14,15], the absorption of UV light [16,17] and the scavenging of hydroxyl radicals [18].

**Figure 1:**
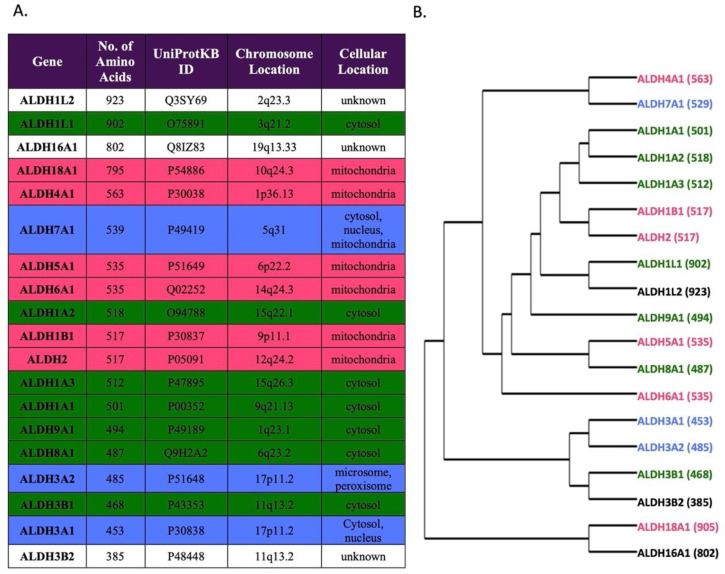
Summary of human ALDH Isozymes. (**A**) Table outlining all 19 human ALDH isozymes with the number of amino acid residues, UniProtKB accession number, chromosomal location, and cellular location listed. (**B**) Phylogenetic tree showing the evolution of human ALDH isozymes with the number of amino acid residues shown in parenthesis (modified from Koppaka et al. (2012) [35]). The color denotes the cellular localization and corresponds between the phylogenetic tree and table as follows: pink, mitochondria; green, cytosol; blue, multiple compartments; no color, unknown location.

The human genome contains 19 functional protein-coding ALDH genes and 3 pseudogenes [19,20]. The 19 functional genes are grouped according to sequence homology, phylogeny, and structural features. Figure 1 shows a dendrogram of the 19 ALDH genes together with their amino acid numbers, protein ID (UniProtKB ID) numbers used by gnomAD variant annotation, chromosome location, and the reported subcellular localization for each isozyme. The 19 ALDH isozymes share a structural similarity. All active ALDH enzymes exist in either a dimeric or tetrameric form containing a catalytic domain, a cofactor (NAD^+^ or NADP^+^)-binding domain and an oligomerization domain [21]. The length of the monomer of the ALDH enzymes ranges from 385 amino acids (ALDH3B2) to 923 amino acids (ALDH1L2) (Figure 1). The 19 ALDH genes are scattered throughout the human genome except for the two pairs of closely related ALDH3A1/ALDH3A2 and ALDH3B1/ALDH3B2 that arose by tandem duplication and reside within <500 kb of each other on chromosome 17p11.2 and chromosome 11q13, respectively. There are no ALDH genes located on the X-chromosome. In addition, the only intron-less ALDH1B1 gene arose by a transposable element-like integration of the ALDH2 gene [22,23]. Both ALDH2 and ALDH1B1 reside within the mitochondria, have the same number of amino acids (517), and share 72% identity in amino acid sequences [22].

The alignment of the 19 human ALDH amino acid sequences has been published [24]. ALDH isozymes share conserved and key features of having Cys-319 (numbering based on the human ALDH2 protein in gnomAD), Glu-285, Gly-316, and Asn-186 as essential amino acid residues for catalysis [25,26], and Gly-262 and Gly-267 for cofactor NAD(P)^+^ binding [27]. Mutations in the ALDH gene family, which results in a loss of function or deficiency of the ALDH enzyme, often lead to accumulation of toxic and reactive aldehydes, inactivation of cellular functions, interruption of normal metabolic pathways, and human diseases [28]. For example, mutations in ALDH2 increase the risk of upper aerodigestive cancer with alcohol consumption [29]. Mutations in ALDH3A2 cause Sjogren–Larsson syndrome [30]. Mutations in ALDH4A1 cause type II hyperprolinemia and result in mental retardation and convulsion [31,32]. Mutations in ALDH5A1 cause 4-hydroxybutyric aciduria [33], and mutations in ALDH7A1 cause pyridoxine-responsive epilepsies [34]. However, the physiological function or the consequences of mutation in ALDH isozymes remain unknown, such as ALDH3B2 and ALDH16A1, which have unknown preferred substrates, and their physiological role is unclear [35].

Using KD4v, a 3D mapping software for the association between known human disease phenotypes and protein variants, Christy and Doss compiled deleterious properties of Single Amino acid Polymorphisms (SAPs) of 19 ALDH genes [36]. In that study, 16 experimentally proved disease-causing SAPs from the ALDH gene family were described and validated. In addition to the ALDH isozymes and diseases mentioned above, the study further compiled the association of ALDH1A1 (A151S, I157T) with congenital heart disease, ALDH1A3 (R89C) with recessive anophthalmia and microphthalmia, ALDH1B1 (A86V) with alcohol-induced hypersensitivity, ALDH1L1 (D793G) with Hodgkin’s lymphoma, ALDH6A1 (R535C, G466R) with dysmyelination and transient methylmalonic aciduria, ALDH16A1 (P527R) with gout and hereditary spastic paraplegias, and ALDH18A1 (R84Q) with urea cycle defects.

With the availability of many large human genome databases and information on human DNA variations, it is clear that genetic variants within the 19 ALDH gene family and their association with the disease should be further explored. The Genome Aggregation Database (gnomAD) is publicly available at https://gnomad.broadinstitute.org/ (accessed on 1 June 2021) [37]. The gnomAD v2.1.1 data set (GRCh37/hg19) is a diverse and comprehensive global human genome database comprised of 125,748 exome sequences and 15,708 whole-genome sequences from various disease-specific and population genetic studies and breaks down the genomic information by the ancestry of the individuals into seven major ethnic groups. Genetic variations compiled in gnomAD include missense variants, loss-of-function variants such as frameshift, start gain/loss, stop gain/loss, and splice donor/acceptor that may cause changes in the primary sequence of the encoded protein [37]. GnomAD also collects synonymous variants, intron variants, and variants in the 3’ and 5’ untranslated regions. These variants do not involve structural changes in the protein but may still cause phenotypic changes in gene expression level, mRNA stability, and translation efficiency of the gene product. Major findings from this aggregated global human genetic information have been published by the gnomAD group, such as a structural variant reference map [38], a transcript variant expression tool [39], assessing drug targets through loss-of-function variants [40], analyzing the impact of multi-nucleotide variants [41], the identification of LRRK2 for therapeutic validation [42], and a reference map of protein structural variants [43].

The purpose of our study is to survey the landscape of human genetic variation within the 19 ALDH multigene family and to provide it as a genetic tool for researchers and clinicians who are interested in studying the structure-function relationship and genetic variations in ALDH that may cause abnormal aldehyde metabolism, accumulation of aldehyde toxicity, and their related diseases. We compiled all relatively common ALDH variants with an allele frequency of ≥0.1% (i.e., more than one carrier per 500 individuals) in any of the 19 ALDH genes from gnomAD. We also categorized these common variants according to the nature of the mutation (Table 1; Tables S1–S19), ethnicity information (European Finnish, East Asian, Latino, European Non-Finnish, South Asian, Ashkenazi Jewish, or Other), and distribution of allele frequency in each of the ethnic groups. Since genetic variations are highly ethnicity-associated, each genetic variant can exhibit a large range of variation in frequency and prevalence, depending on the geographical location, when the variant arose, and natural selection on the mutation. For example, the ALDH2 rs671 (E504K) variant, which originated in southeastern China about 2000–3000 years ago [44] and resulted in ALDH2 enzyme deficiency, is well known to cause the Asian-specific alcohol flushing reaction [45]. As expected, we found that the rs671 (E504K) variant was highly prevalent among the East Asians (25.5%), while almost absent or existed in a very low frequency among other ethnic groups in the world (e.g., 0.002% in non-Finnish Europeans) based on the data recorded in gnomAD. Depending on the nature and the position of the mutations, although some may be benign variants, other ALDH variants are likely to be pathogenic. As current clinical medical practices emphasize individual, group, or race-based health improvement, disease prevention, diagnosis or treatment [46], the data compiled in this publication will certainly contribute to the understanding of aldehyde toxicity and aldehyde metabolism in humans and how specific ALDH variants affect aldehyde metabolism.

## 2. Materials and Methods

The Genome Aggregation Database, gnomAD v2.1.1 (https://gnomad.broadinstitute.org/ [37]) (accessed on 1 June 2021) was used to search all 19 ALDH genes using gene names as shown in Figure 1. Raw data of all recorded variants for each ALDH gene in gnomAD was retrieved and exported as a Comma Separated Values (CSV) file then converted into an individual Excel file (Microsoft Excel version 16.52, Redmond WA, USA)for each variant allele frequency calculation, filtering, sorting, and rearrangement. Data and features captured were chromosomal position, rsID (Reference SNP cluster ID), HGVS (Humane Genome Variation Society) nomenclature consequence, VEP (Variant Effect Predictor) annotation, ethnicity, and allele counts in each of the 7 ethnic groups. We calculated allele frequencies by dividing the allele count of each identified variant by the total allele count compiled in the database for the 7 different ethnic groups. The 7 representative ethnic groups in gnomAD are Non-Finnish Europeans (64,603 individuals), Latinos (17,720), South Asians (15,308), Finnish (12,562), African/African American (12,487), East Asians (9977), Ashkenazi Jews (5185), and others (3614 individuals) [37]. The data were first filtered for common variants, which were defined by the criteria of allele frequency ≥0.1% (or 1 carrier per 500 individuals) in any of the 7 ethnic groups recorded in gnomAD. The data sets were then sorted by VEP annotation to include 5’ UTR variants, 3’ UTR variants, intron variants, frameshift variants, missense variants, synonymous variants, splice acceptor variants, splice donor variants, splice region variants, start lost variants, stop gain variants, and stop lost variants. A resulting joint table with all variables analyzed was created for each of the 19 ALDH genes (Tables S1–S19).

For structural and functional prediction of the missense variants, gnomAD also included information based on PolyPhen (Polymorphism Phenotyping) [47], computer-assisted automatic analysis of the possible impact of an amino acid substitution on the structure and function of a human protein, and SIFT (Sorting Intolerant From Tolerant) [48], a program that predicts whether an amino acid substitution would affect protein function. Polyphen/SIFT data for all missense variants were extracted from gnomAD database using the free Chrome internet extension “Web Scraper” (https://webscraper.io/, Version 0.6.1) (accessed on 16 June 2021). In addition, pLOF (Loss-of-Function) variants, defined as variants that were nonsense, frameshift, or had mutations in the canonical splice site (intronic +1, +2, −1, −2) were retrieved from the gnomAD database. All pLOF variants that occur at a frequency of at least 0.1% in any ethnic group were also compiled in our study. Variants that had unknown Polyphen/SIFT data because the software could not make a prediction due to a lack of data were excluded from the analysis. The SIFT predictions of “deleterious” and “deleterious_low_confidence” were combined as “deleterious,” and predictions of “tolerated” and “tolerated_low_confidence” were combined as “tolerated” in our annotation. Polyphen and SIFT predictions were attached to their corresponding SNP (Tables S1–S19). We consider variants that are predicted by Polyphen and SIFT to be “probably damaging” and “deleterious,” respectively, are predicted to be more damaging than variants predicted by both algorithms to be “benign” and “tolerated,” respectively.

Gene constraint analysis on synonymous variants, missense variants, and pLOF for each ALDH gene was carried out by compiling the data of Expected Variant Counts (Exp. SNVs), Observed Variant Counts (Obs. SNVs), and Constraint Metrics from gnomAD. The Constraint Metrics was expressed as a ratio between the Observed SNVs and Expected SNVs values (o/e ratio) with the display of the 90% confidence interval (CI) for each of the o/e value. A gene is considered to be under much stronger selection pressure for that class of variation when it has a lower o/e value than a gene that has a higher o/e value.

## 3. Results

### 3.1. Analysis of Common ALDH Variants

**Figure 2:**
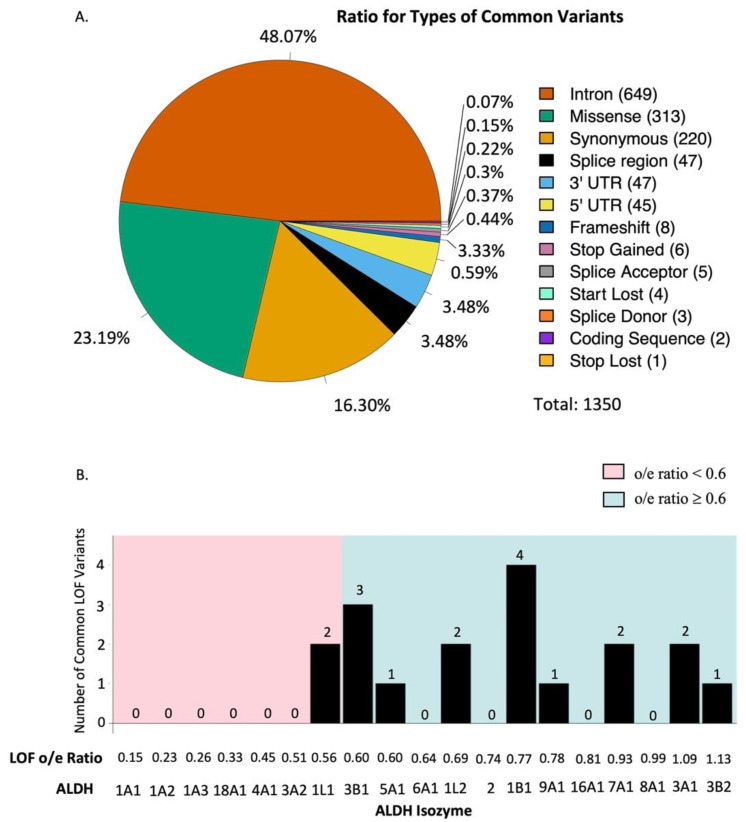
Summary of common variants of ALDH isozymes. (**A**) Ratio of the type of common variants present in the 19 human ALDH isozymes. (**B**) Number of common loss-of-function variants in all 19 ALDH isozymes. The isozymes are listed in ascending order of the loss of function o/e ratio.

The majority of the variants in the ALDH multigene family in the gnomAD database are rare or occur in low frequency, thus likely representing random mutations in the population. We filtered out the rare variants and only list fairly common variants (≥0.1% in any given ethnic group) in any one of the ethnic groups. Since each person carries two alleles for each gene, an allele frequency of ≥0.1% represents a variant that can be found in at least 1 carrier from 500 individuals in any given ethnic group. Tables S1–S19 show detailed information of these common variants in each of the 19 ALDH genes. For the variants of each ALDH gene, we compiled their chromosomal position, RSID (Reference SNP cluster ID), HGVS (Human Genome Variation Society) nomenclature consequence (i.e., nucleotide change, amino acid change), VEP (Variant Effect Predictor) annotation, ethnicity information, and allele frequencies for each of the seven ethnic groups. We analyzed which type of variants were most common across all 19 ALDH genes (i.e., 5’ and 3’ UTR, frameshift, intron, missense, splice donor/acceptor, splice region, start gain/loss, stop gain/loss, and synonymous variants) to determine which types of variants were most common across the ALDH isozymes. In total, we found 1350 different common ALDH variants that met the criteria of ≥0.1% allele frequency in any of the seven ethnic groups. Table 1 summarizes the VEP annotation of these 1350 variants for each of the 19 ALDH genes. The most common variants for the 19 ALDH genes were intron variants (649), which accounted for 48% of all 1350 common variants, followed by missense (313), synonymous (220), splice region (47), 3’-UTR (47), 5’-UTR (45), frameshift (8), stop gain (6), splice acceptor (5), start lost (4), splice donor (3), undefined coding sequence (2), and stop lost (1) variants (Figure 2A).

For the 313 common ALDH missense variants, we also collected the information on structural, functional prediction of these single amino acid substitutions from gnomAD. We used PolyPhen (Polymorphism Phenotyping) and SIFT (Sorting Intolerant From Tolerant) information collected by gnomAD to predict the consequences of a missense mutation to its protein structure and function. The predicted consequences of single amino acid change were classified by categories of “probably damaging,” “possibly damaging,” “benign,” “null” or “unknown” according to its predicted severity for PolyPhen and “deleterious,” “tolerated,” or “null” according to SIFT. For example, the East Asian ALDH2 rs671 E504K missense variant is well characterized for its clinical phenotype of alcohol flushing [29] and the mutation is known to affect dimer-dimer formation and co-enzyme binding with a >90% reduction of enzyme activity [49]. This missense variant was classified as “probably damaging” and “deleterious,” respectively, based on PolyPhen and SIFT prediction in gnomAD. The impact on protein structure and function based on PolyPhen and SIFT prediction are also listed in Tables S1–S19 for all common missense variants of each of the ALDH variants. Among the 313 ALDH missense variants, 86 were categorized as “probably damaging,” 53 were “possibly damaging,” 165 were “benign,” 1 was “null,” and 8 were “unknown” according to Polyphen prediction. A total of 166 missense variants were categorized as “deleterious,” 141 were “tolerated,” and 6 variants were “null” according to SIFT prediction. Our analysis identified 81 common missense variants that we considered as having the most likely predicted damaging effect on the protein structure and function with simultaneously classification of “probably damaging” by PolyPhen and “deleterious” by SIFT within the 19 ALDH gene family. For example, four missense variants of ALDH2 were predicted to be “probably damaging” and “deleterious” and could contribute to ethanol sensitivity in East Asians, South Asians, and Finnish-Europeans, which are the ethnicities that carry those variants most often (Table S7). Additionally, 10 common missense variants are found in ALDH3B2 that are predicted to be “probably damaging” and “deleterious” (Table S11). Screening populations who harbor these variants for associations to human disease may help elucidate the currently unknown physiological function of this enzyme [35]. These variants warrant further investigation as they may be associated with decreased protein activity and negatively impact human health. 

### 3.2. Gene Constraint Analysis

The 19 human ALDH genes have evolved over time with overlapping substrates for enzymatic detoxification of endogenous and exogenous aldehydes [20,50]. Surveying different types of mutations in these genes is a valid strategy to understand how selection forces have been exerted on the structure and function of this multigene family. Some of the ALDH isozymes may serve a more essential and indispensable developmental and physiological function, whereas other ALDH isozyme may have evolved more recently and serve a more redundant function. It is expected that, in human populations, fewer numbers of viable mutations will be tolerated in those ALDH genes that are more essential than those that are more dispensable. The number and type of variants in each ALDH gene is, therefore, a good indicator of the functional importance of the ALDH isozymes. GnomAD uses constraint score to measure how tolerant a gene is to different types of mutations (e.g., synonymous, missense, and loss-of-function). It uses a computer mutational model that takes into account sequence context, coverage, and methylation to calculate expected counts of mutation for a particular gene. The constraint score is then derived as the ratio of the actual observed/expected (o/e) numbers for a specific type of variant in that gene [51]. An o/e approaching 1.0 is, therefore, indicative of high tolerance (i.e., low constraint) for mutations for the gene and a lower o/e ratio is indicative of strong intolerance for mutations for the gene. For example, for loss-of-function variants, an o/e ratio of zero means the gene is under extreme selection pressure against loss-of-function mutations and that no individuals carrying a loss-of-function allele were ever found in the gnomAD database.

We conducted gene constraint analysis for the 19 ALDH genes by collecting available o/e ratio information from gnomAD for synonymous, missense, and loss-of-function variants (Table 2). For synonymous variants, the o/e ratios of the 19 ALDH genes fell within a relatively moderate range between 0.81 (ALDH3A2) and 1.22 (ALDH1A2), which means that synonymous mutations were quite well tolerated for all the 19 ALDH genes. For missense variants, the o/e ratio of the 19 ALDH genes had a slightly lower range below 1.0, between 0.64–1.07, with ALDH1A3 having the lowest o/e ratio of 0.64 for missense variants. For loss-of-function variants, a significantly wider and lower range of 0.15–1.09 was observed for the 19 ALDH genes. Of note, the three genes in the ALDH1A family, ALDH1A1 (0.15), ALDH1A2 (0.23), and ALDH1A3 (0.26), revealed exceptionally low numbers of observed vs. expected loss-of-function variants. These three genes also ranked with the lowest o/e ratios among the 19 ALDH genes for the missense mutation. In addition, ALDH18A1 (0.33), ALDH4A1 (0.45), ALDH3A2 (0.51), and ALDH1L1 (0.56), also had significantly lower o/e ratio of <0.60 for the loss-of-function variants. It means that structural change or loss-of-function mutations were not well tolerated for these ALDH genes. 

Gene constraint analysis showed the number and type of mutation but did not take into account allele frequency for each of the mutations. From the 1350 common variants, we found 19 potential loss-of-function variants, including 8 frameshift, 6 stop-gain, 4 start-lost, and 1 stop-lost variants. 18 of the 19 potential loss-of-function common variants all clustered within ALDH genes that had a relatively high o/e ratio of ≥0.6 (i.e., well-tolerated genes), except for a common start-lost variant found in ALDH1A2 (o/e = 0.23). Of the 18 common loss-of-function variants across all ALDH genes, 4 were observed in ALDH1B1 (o/e = 0.77), 3 in ALDH3B1 (o/e = 0.60), 2 each in ALDH3A1 (o/e = 1.09), ALDH7A1 (o/e = 0.93), ALDH1L1 (o/e = 0.56), and ALDH1L2 (o/e = 0.69), 1 each in ALDH3B2 (o/e = 1.13), ALDH9A1 (o/e = 0.78), and ALDH5A1 (o/e = 0.60) (Table 2, Figure 2B). Therefore, there was a good agreement within the 19 ALDH gene family between the absence of common loss-of-function variants and high selection force against loss-of-function mutations (i.e., low o/e ratio) of the gene.

## 4. Discussion

The gnomAD database is a human genome database containing sequences from seven representative ethnic groups in the world [37]. Even though the majority of the participants included in the analysis are considered “healthy” subjects, there are many genetic variants with potential clinical consequences that should be further characterized. In this analysis, we focused on the functionally defined and related multigene family of human aldehyde dehydrogenase (ALDH) to compile 1350 common genetic variants for all 19 members of the ALDH gene family from gnomAD to identify further common variants that may impact human health. We focused on the ALDH family because of its impact on several human diseases such as Sjogren–Larsson syndrome [30], type II hyperprolinemia, and mental retardation [31,32]. For example, ALDH7A1 deficiency results in pyridoxine-responsive epilepsies [34], and our analysis identified 13 common missense variants in ALDH7A1, which may also impact patients’ risk of developing a neurological disorder, warranting further investigation. 

The 1350 common genetic variants of ALDH were selected by applying a filter of allele frequency ≥0.1 in any of the seven ethnic groups recorded in gnomAD. Using this approach, we previously identified and characterized five new, common non-East Asia ALDH2 missense variants from the ExAc human genome database [52], in addition to the well-known East Asian ALDH2 E504K variant [53]. For example, we found a common P92T missense variant and an R338W missense variant with 2.5% and 1.2% allele frequencies in the Latino and Finnish populations, respectively. Site-directed mutagenesis and cell culture studies showed that these new ALDH2 missense variants also had reduced enzyme activity and were more susceptible to aldehyde toxicity [53]. Based on these results, we anticipate that the alcohol flushing reaction and susceptibility to aldehyde toxicity due to ALDH2 enzyme deficiency in different non-East Asian ethnic groups may exist and are likely more common than previously thought. Identifying human subjects carrying these new variants with their associated phenotypes can, therefore, be studied by this approach of data mining using a large human genome database. 

The ExAc human genome database with additional human genetic cohorts has now been merged as a single and larger gnomAD database [37], which is used in this study. In addition to missense mutations, we expanded our search to include other types of mutations categorized by gnomAD. Besides ALDH2, we also compiled with all other 18 functional ALDH genes to complete a whole list of 1350 common variants for the ALDH multigene family. The cut-off filtering criteria of ≥0.1% allele frequency was chosen arbitrarily. This arbitrary cut-off value may have missed important but rarer genetic variants that could have clinical or health consequences. On the other hand, if we were to apply a less stringent cut-off value of ≥1% allele frequency or ≥1 carrier per 50 individuals, the number of common variants for the 19 ALDH gene family would be reduced from 1350 to 611, which may exclude clinically relevant variants circulating in the human population. For example, this cutoff would exclude the ALDH2 variant I41V (Table S7), which we previously characterized as less active than the wild type and increases sensitivity to ethanol [53].

Among the 1350 common ALDH variants compiled in this study, we observed a positive correlation between the size of the protein-coding region (exons) of a gene and the number of common variants in each gene. However, the number of variants in each gene is also dependent on the constraint of the gene imposed by selection force on its function, as reflected by the o/e ratio (Table 2). For example, the larger ALDH1L2, ALDH1L1, and ALDH16A1 genes, which encode a polypeptide with ~800–920 amino acids, had about 110–135 common variants, whereas the smaller ALDH genes such as ALDH3A1, ALDH3B1, and ALDH3B2, which encode a polypeptide with 380–470 amino acids only had about 78–86 common variants (Figure 1, Table 1). Exceptions were observed in ALDH18A1, which had ~800 amino acids, similar to ALDH1L1, ALDH1L2, and ALDH16A1, but only had 61 common variants, or in ALDH3A2, which had ~480 amino acids, similar to ALDH3A1, ALDH3B1, ALDH3B2, but only had 34 common variants. 

The o/e ratio for the number of loss-of-function variants serves as a good indicator on how well null mutations were tolerated in the human population. The three members of the ALDH1A subfamily, ALDH1A1, ALDH1A2, and ALDH1A3 had the lowest o/e ratios (0.15–0.26) among the 19 ALDH gene members, in contrast to ALDH3A1 and ALDH3B2, which had the highest and most tolerable o/e ratios (1.09–1.13) for loss-of-function constraint. This implies that the ALDH1A1, ALDH1A2, and ALDH3A1 genes may have a more essential and irreplaceable physiological function than the ALDH3A1 and ALDH3B2 genes in humans. Gene constraint analysis is, therefore, a useful ranking tool to predict the degree of redundancy for multigene families that may have members with overlapping functions. 

The 19 ALDH genes have evolved with different substrate specificity, subcellular location, tissue distribution, and, perhaps, overlapping physiological functions. Mutations and genetic polymorphisms in several ALDH genes have been linked to known human diseases, but the exact function and substrate specificity of many ALDH genes remain unknown, such as ALDH3B2 [35]. Traditional research on the human genetic disease has been practiced by characterizing a disease followed by steps of mapping, molecular cloning, or DNA sequencing to define its underlying genetic causes. The availability of large human genomic sequence data in different races, countries, and geographic regions now makes it possible to follow genetic variants in subgroups of a population-based on their chromosomal position, the nature of the mutation, structural/functional prediction (such as PolyPhen and SIFT), allelic prevalence and ethnicity of the mutation for the study of genetic epidemiology, clinical observation and disease prevention, which has begun for some cytochrome P450 oxidizing enzymes [54]. For example, the 81 missense variants that we identified with the simultaneous classification of “probably damaging” by PolyPhen and “deleterious” by SIFT are likely to have reduced ALDH function. In conjunction with specific information on ethnicity and allele frequency of these variants, this information can be used in a clinical setting by medical doctors to screen patients for common ALDH alleles that may be risk factors for disease. This will be particularly helpful as our analysis identifies which ethnicity is most likely to carry certain variants and may aid physicians in identifying risk alleles if they are aware of their patient’s ethnicity. We hope our analysis will inspire further epidemiological studies to determine whether these common ALDH variants increase the risk for a specific disease (e.g., cancer), as is the case for ALDH2 [29]. Additionally, analyzing the impact of common ALDH variants on their respective activity will aid researchers in identifying critical structural domains of the ALDH tetramers for their function, which may assist in identifying the function of ALDH isozymes across animals, plants, and bacteria. Additionally, understanding the impact of these variants will provide insight into the function of endogenous and exogenous aldehydes and how they impact human health. Furthermore, sequencing patients for these common variants may identify patient populations that could benefit from existing small molecules such as Alda-1 and Alda-64 [53] to activate variant ALDH isozymes to enhance protein activity and mitigate disease risk. 

## Figures and Tables

**Table 1:** Summary of 1350 common variants from 19 human ALDH genes. The number of variants of 13 different types of DNA mutations in each gene are listed based to their Variant Effect Predictor (VEP) annotations.

Gene	No. of Amino Acid	No. of Variants ≥0.1%	Intron	Missense	Synonymous	Splice Region	3’ UTR	5’ UTR	Frameshift	Stop Gained	Splice Acceptor	Start Lost	Splice Donor	Coding Sequence	Stop Lost
ALDH1A1	501	49	32	5	5	4	1	1	0	0	1	0	0	0	0
ALDH1A2	518	68	45	6	8	2	1	5	0	0	0	1	0	0	0
ALDH1A3	512	70	33	10	17	3	5	2	0	0	0	0	0	0	0
ALDH1B1	517	36	0	19	10	0	3	0	3	1	0	0	0	0	0
ALDH1L1	902	124	63	26	19	7	4	1	0	2	1	0	1	0	0
ALDH1L2	923	109	72	19	9	2	3	2	0	2	0	0	0	0	0
ALDH2	517	47	26	9	9	0	1	1	0	0	0	0	1	0	0
ALDH3A1	453	86	42	24	12	0	4	1	1	0	1	1	0	0	0
ALDH3A2	485	34	15	9	6	2	0	1	0	0	1	0	0	0	0
ALDH3B1	468	78	33	14	15	2	8	0	3	0	1	0	0	2	0
ALDH3B2	385	84	35	32	8	1	3	4	0	1	0	0	0	0	0
ALDH4A1	563	102	51	23	17	2	3	6	0	0	0	0	0	0	0
ALDH5A1	535	59	19	20	9	4	2	4	0	0	0	0	0	0	1
ALDH6A1	535	36	17	6	4	6	2	1	0	0	0	0	0	0	0
ALDH7A1	539	74	44	13	9	1	1	4	1	0	0	1	0	0	0
ALDH8A1	487	35	6	10	10	4	1	4	0	0	0	0	0	0	0
ALDH9A1	494	62	22	22	7	2	1	6	0	0	0	1	1	0	0
ALDH16A1	802	136	63	35	33	2	1	2	0	0	0	0	0	0	0
ALDH18A1	795	61	31	11	13	3	3	0	0	0	0	0	0	0	0
**Total**		**1350**	**649**	**313**	**220**	**47**	**47**	**45**	**8**	**6**	**5**	**4**	**3**	**2**	**1**

**Table 2:** Gene constraint analysis of synonymous variants, missense variants, and loss-of-function variants of the 19 human ALDH genes. Exp. SNVs (Expected Variant Counts); Obs. SNVs (Observed Variant Counts). The constraint metrics (o/e ratio) is defined as the ratio between Obs. SNPs and Exp. SNVs. The parenthesis shows the 90% confidence interval (CI) for each of the o/e value. Genes that are less tolerant to certain class of genetic variants (value of o/e ratio <0.60) are indicated in bold faces.

			Synonymous Variants			Missense Variants			Loss of Function Variants		
Gene	UniProtKB ID	No. of Amino Acid	Exp. SNVs	Obs. SNVs	Constraint Metrics (o/e Ratio)	Exp. SNVs	Obs. SNVs	Constraint Metrics (o/e Ratio)	Exp. SNVs	Obs. SNVs	Constraint Metrics (o/e Ratio)
ALDH1A1	P00352	501	101.6	106.0	1.04 (0.89–1.23)	276.4	199.0	0.72 (0.64–0.81)	27.3	4.0	**0.15 (0.07–0.34)**
ALDH1A2	O94788	518	105.2	128.0	1.22 (1.05–1.41)	283.1	215.0	0.76 (0.68–0.85)	26.5	6.0	**0.23 (0.12–0.45)**
ALDH1A3	P47895	512	123.0	122.0	0.99 (0.85–1.15)	301.2	192.0	0.64 (0.57–0.72)	23.2	6.0	**0.26 (0.14–0.51)**
ALDH18A1	P54886	795	165.8	158.0	0.95 (0.84–1.09)	441.2	325.0	0.74 (0.67–0.81)	39.8	13.0	**0.33 (0.21–0.52)**
ALDH4A1	P30038	563	156.0	150.0	0.96 (0.84–1.10)	354.5	357.0	1.01 (0.92–1.10)	28.9	13.0	**0.45 (0.29–0.71)**
ALDH3A2	P51648	485	104.9	85.0	0.81 (0.68–0.97)	277.0	245.0	0.88 (0.80–0.98)	23.6	12.0	**0.51 (0.33–0.82)**
ALDH1L1	O75891	902	235.3	241.0	1.02 (0.92–1.14)	552.0	512.0	0.93 (0.86–1.00)	45.0	25.0	**0.56 (0.40–0.78)**
ALDH3B1	P43353	468	128.5	130.0	1.01 (0.88–1.17)	306.2	259.0	0.85 (0.76–0.94)	18.3	11.0	0.60 (0.38–0.99)
ALDH5A1	P51649	535	119.5	112.0	0.94 (0.80–1.10)	289.1	254.0	0.88 (0.79–0.97)	23.4	14.0	0.60 (0.40–0.94)
ALDH6A1	Q02252	535	103.6	87.0	0.84 (0.70–1.00)	292.3	242.0	0.83 (0.74–0.92)	29.5	19.0	0.64 (0.45–0.94)
ALDH1L2	Q3SY69	923	183.0	167.0	0.91 (0.80–1.04)	500.3	414.0	0.83 (0.76–0.90)	49.1	34.0	0.69 (0.53–0.92)
ALDH2	P05091	517	134.9	143.0	1.06 (0.92–1.22)	316.1	250.0	0.79 (0.71–0.88)	24.4	18.0	0.74 (0.51–1.09)
ALDH1B1	P30837	517	140.5	133.0	0.95 (0.82–1.09)	321.5	333.0	1.04 (0.95–1.13)	13.0	10.0	0.77 (0.47–1.30)
ALDH9A1	P49189	494	107.7	119.0	1.10 (0.95–1.29)	292.0	312.0	1.07 (0.97–1.17)	25.5	20.0	0.78 (0.55–1.14)
ALDH16A1	Q8IZ83	802	228.6	228.0	1.00 (0.89–1.11)	503.3	530.0	1.05 (0.98–1.13)	43.1	35.0	0.81 (0.62–1.08)
ALDH7A1	P49419	539	105.6	108.0	1.02 (0.87–1.20)	295.8	278.0	0.94 (0.85–1.04)	37.4	35.0	0.93 (0.71–1.24)
ALDH8A1	Q9H2A2	487	122.0	115.0	0.94 (0.81–1.10)	291.8	268.0	0.92 (0.83–1.02)	18.2	18.0	0.99 (0.68–1.46)
ALDH3A1	P30838	453	128.6	137.0	1.07 (0.93–1.23)	287.1	273.0	0.95 (0.86–1.05)	22.0	24.0	1.09 (0.79–1.53)
ALDH3B2	P48448	385	114.9	123.0	1.07 (0.92–1.24)	250.2	256.0	1.02 (0.92–1.14)	16.0	18.0	1.13 (0.78–1.66)

## Data Availability

All data are available with the manuscript and at https://gnomad.broadinstitute.org (accessed 1 June 2021).

## Supplementary Materials

The following are available at https://www.mdpi.com/article/10.3390/biom11101423/s1, Table S1: Common ALDH1A1 variants with allele frequency >0.1% in at least one the seven ethnic groups. The variants are arranged according to their chromosomal position, RSID, HGVS Consequence, Variant Effect Predictor (VEP) annotation and ethnicity. PolyPhen and SIFT predictions are also listed for missense variants. Table S2: Common ALDH1A2 variants with allele frequency >0.1% in at least one the seven ethnic groups. The variants are arranged according to their chromosomal position, RSID, HGVS Consequence, Variant Effect Predictor (VEP) annotation and ethnicity. PolyPhen and SIFT predictions are also listed for missense variants. Table S3: Common ALDH1A3 variants with allele frequency >0.1% in at least one the seven ethnic groups. The variants are arranged according to their chromosomal position, RSID, HGVS Consequence, Variant Effect Predictor (VEP) annotation and ethnicity. PolyPhen and SIFT predictions are also listed for missense variants. Table S4: Common ALDH1B1 variants with allele frequency >0.1% in at least one the seven ethnic groups. The variants are arranged according to their chromosomal position, RSID, HGVS Consequence, Variant Effect Predictor (VEP) annotation and ethnicity. PolyPhen and SIFT predictions are also listed for missense variants. Table S5: Common ALDH1L1 variants with allele frequency >0.1% in at least one the seven ethnic groups. The variants are arranged according to their chromosomal position, RSID, HGVS Consequence, Variant Effect Predictor (VEP) annotation and ethnicity. PolyPhen and SIFT predictions are also listed for missense variants. Table S6: Common ALDH1L2 variants with allele frequency >0.1% in at least one the seven ethnic groups. The variants are arranged according to their chromosomal position, RSID, HGVS Consequence, Variant Effect Predictor (VEP) annotation and ethnicity. PolyPhen and SIFT predictions are also listed for missense variants. Table S7: Common ALDH2 variants with allele frequency >0.1% in at least one the seven ethnic groups. The variants are arranged according to their chromosomal position, RSID, HGVS Consequence, Variant Effect Predictor (VEP) annotation and ethnicity. PolyPhen and SIFT predictions are also listed for missense variants. Table S8: Common ALDH3A1 variants with allele frequency >0.1% in at least one the seven ethnic groups. The variants are arranged according to their chromosomal position, RSID, HGVS Consequence, Variant Effect Predictor (VEP) annotation and ethnicity. PolyPhen and SIFT predictions are also listed for missense variants. Table S9: Common ALDH3A2 variants with allele frequency >0.1% in at least one the seven ethnic groups. The variants are arranged according to their chromosomal position, RSID, HGVS Consequence, Variant Effect Predictor (VEP) annotation and ethnicity. PolyPhen and SIFT predictions are also listed for missense variants. Table S10: Common ALDH3B1 variants with allele frequency >0.1% in at least one the seven ethnic groups. The variants are arranged according to their chromosomal position, RSID, HGVS Consequence, Variant Effect Predictor (VEP) annotation and ethnicity. PolyPhen and SIFT predictions are also listed for missense variants. Table S11: Common ALDH3B2 variants with allele frequency >0.1% in at least one the seven ethnic groups. The variants are arranged according to their chromosomal position, RSID, HGVS Consequence, Variant Effect Predictor (VEP) annotation and ethnicity. PolyPhen and SIFT predictions are also listed for missense variants. Table S12: Common ALDH4A1 variants with allele frequency >0.1% in at least one the seven ethnic groups. The variants are arranged according to their chromosomal position, RSID, HGVS Consequence, Variant Effect Predictor (VEP) annotation and ethnicity. PolyPhen and SIFT predictions are also listed for missense variants. Table S13: Common ALDH5A1 variants with allele frequency >0.1% in at least one the seven ethnic groups. The variants are arranged according to their chromosomal position, RSID, HGVS Consequence, Variant Effect Predictor (VEP) annotation and ethnicity. PolyPhen and SIFT predictions are also listed for missense variants. Table S14: Common ALDH6A1 variants with allele frequency >0.1% in at least one the seven ethnic groups. The variants are arranged according to their chromosomal position, RSID, HGVS Consequence, Variant Effect Predictor (VEP) annotation and ethnicity. PolyPhen and SIFT predictions are also listed for missense variants. Table S15: Common ALDH7A1 variants with allele frequency >0.1% in at least one the seven ethnic groups. The variants are arranged according to their chromosomal position, RSID, HGVS Consequence, Variant Effect Predictor (VEP) annotation and ethnicity. PolyPhen and SIFT predictions are also listed for missense variants. Table S16: Common ALDH8A1 variants with allele frequency >0.1% in at least one the seven ethnic groups. The variants are arranged according to their chromosomal position, RSID, HGVS Consequence, Variant Effect Predictor (VEP) annotation and ethnicity. PolyPhen and SIFT predictions are also listed for missense variants. Table S17: Common ALDH9A1 variants with allele frequency >0.1% in at least one the seven ethnic groups. The variants are arranged according to their chromosomal position, RSID, HGVS Consequence, Variant Effect Predictor (VEP) annotation and ethnicity. PolyPhen and SIFT predictions are also listed for missense variants. Table S18: Common ALDH16A1 variants with allele frequency >0.1% in at least one the seven ethnic groups. The variants are arranged according to their chromosomal position, RSID, HGVS Consequence, Variant Effect Predictor (VEP) annotation and ethnicity. PolyPhen and SIFT predictions are also listed for missense variants. Table S19: Common ALDH18A1 variants with allele frequency >0.1% in at least one the seven ethnic groups. The variants are arranged according to their chromosomal position, RSID, HGVS Consequence, Variant Effect Predictor (VEP) annotation and ethnicity. PolyPhen and SIFT predictions are also listed for missense variants.
